# Failure Of Hearing Acquisition in Mice With Reduced Expression of Connexin 26 Correlates With the Abnormal Phasing of Apoptosis Relative to Autophagy and Defective ATP-Dependent Ca^2+^ Signaling in Kölliker’s Organ

**DOI:** 10.3389/fncel.2022.816079

**Published:** 2022-03-03

**Authors:** Lianhua Sun, Dekun Gao, Junmin Chen, Shule Hou, Yue Li, Yuyu Huang, Fabio Mammano, Jianyong Chen, Jun Yang

**Affiliations:** ^1^Department of Otorhinolaryngology-Head and Neck Surgery, Xinhua Hospital, Shanghai Jiaotong University School of Medicine, Shanghai, China; ^2^Ear Institute, Shanghai Jiaotong University School of Medicine, Shanghai, China; ^3^Shanghai Key Laboratory of Translational Medicine on Ear and Nose Diseases, Shanghai, China; ^4^Department of Physics and Astronomy “G. Galilei”, University of Padua, Padua, Italy; ^5^Department of Biomedical Sciences, Institute of Biochemistry and Cell Biology, Italian National Research Council, Monterotondo, Italy

**Keywords:** apoptosis, ATP, autophagy, Ca^2+^, development, deafness

## Abstract

Mutations in the *GJB2* gene that encodes connexin 26 (Cx26) are the predominant cause of prelingual hereditary deafness, and the most frequently encountered variants cause complete loss of protein function. To investigate how Cx26 deficiency induces deafness, we examined the levels of apoptosis and autophagy in *Gjb2*^loxP/loxP^; *ROSA26*^CreER^ mice injected with tamoxifen on the day of birth. After weaning, these mice exhibited severe hearing impairment and reduced Cx26 expression in the cochlear duct. Terminal deoxynucleotidyl transferase dUTP nick end labeling (TUNEL) positive cells were observed in apical, middle, and basal turns of Kölliker’s organ at postnatal (P) day 1 (P1), associated with increased expression levels of cleaved caspase 3, but decreased levels of autophagy-related proteins LC3-II, P62, and Beclin1. In Kölliker’s organ cells with decreased Cx26 expression, we also found significantly reduced levels of intracellular ATP and hampered Ca^2+^ responses evoked by extracellular ATP application. These results offer novel insight into the mechanisms that prevent hearing acquisition in mouse models of non-syndromic hearing impairment due to Cx26 loss of function.

## Introduction

The sense of hearing originates in a portion of the cochlear sensory epithelium, the organ of Corti, which comprises two types of mechanosensory hair cells, the inner and outer hair cells (IHCs and OHCs), which do not express connexins, and at least six types of associated supporting cells, all of which express connexins. Connexin 26 (Cx26, encoded by the *GJB2* gene) and the closely related connexin 30 (Cx30, encoded by *GJB6*) are the prevailing isoforms expressed in non-sensory cells of both the epithelial and connective tissue of the developing and mature cochlea (Forge et al., 2003; Cohen-Salmon et al., 2005).

*GJB2* mutations are a frequent cause of both syndromic and non-syndromic congenital deafness, with an unusually high carrier rate for truncating mutations among hearing-impaired individuals (Chan and Chang, 2014; Del Castillo and Del Castillo, 2017). Connexin proteins form large-pore hexameric plasma membrane channels, termed hemichannels, which may dock head-to-head in the extracellular space to form intercellular gap junction channels (Laird and Lampe, 2022). Interruption of the potassium ion recycling pathway *via* gap junction systems in the mammalian cochlea has been postulated as the cause of hereditary non-syndromic deafness (Kikuchi et al., 2000). However, this hypothesis lacks experimental proof and is contradicted by different studies (Beltramello et al., 2005; Jagger and Forge, 2015; Zhao, 2017). In contrast, the available evidence from mouse models points to a fundamental role played by connexins, particularly connexin hemichannels, during the crucial phases of postnatal cochlear development that lead to hearing acquisitions; reviewed in (Mammano, 2013, 2019).

In the developing rodent cochlea, the sensory epithelium is subdivided into a cellularly dense medial domain named Kölliker’s organ and a less dense lateral domain, the lesser epithelial ridge (LER), separated by a central prosensory region that contains the precursors of the organ of Corti (Lim and Anniko, 1985; Lim and Rueda, 1992; Driver and Kelley, 2020). Kölliker’s organ is one of the earliest structures in the inner ear, recognizable from embryonic (E) day 14 (E14) to postnatal (P) day 12–14 (P12–14, P0 indicates the day of birth), which marks the onset of hearing function that reaches adult-level auditory thresholds by the third postnatal week (Ehret, 1977).

In the pre-hearing phase of mouse cochlear development, Kölliker’s organ cells release ATP periodically through connexin hemichannels (Schutz et al., 2010; Rodriguez et al., 2012; Xu et al., 2017; Zorzi et al., 2017; Mazzarda et al., 2020) to activate purinergic receptors in the surrounding cells, depolarize the hair cells and activate auditory nerve fibers (Tritsch et al., 2007; Wang and Bergles, 2015; Johnson et al., 2017; Eckrich et al., 2018; Ceriani et al., 2019). Spontaneous Ca^2+^ activity in the mouse postnatal cochlea wanes as the sensory epithelium and its innervation pattern mature, in parallel with intense remodeling which leads to the formation of the inner sulcus in place of the degenerated Kölliker’s organ and outer sulcus in place of the LER (Lim and Anniko, 1985; Lim and Rueda, 1992; Driver and Kelley, 2020).

Ca^2+^ signaling, autophagic and apoptotic processes are key to this crucial remodeling phase (La Rovere et al., 2016; Bootman et al., 2018; Mammano and Bortolozzi, 2018; Zhou et al., 2020; Soundarrajan et al., 2021). Recent work examined Kölliker’s organ morphological changes with autophagy and apoptosis markers between P1 and P14 and showed that: (i) autophagy is present and associated closely with the remodeling that leads to Kölliker’s organ degeneration; (ii) Kölliker’s organ cells are digested and absorbed by autophagy before apoptosis occurs (Hou et al., 2019). Here, we extended those studies by investigating the complex interplay between apoptosis, autophagy, ATP, and Ca^2+^ signaling in connection with the failure of hearing acquisition induced by Cx26 deficiency in a mouse model of non-syndromic deafness.

## Materials and Methods

### Animals

*Gjb2*^loxP/loxP^ mice (Cohen-Salmon et al., 2002) and ROSA26^CreER^ mice (Vooijs et al., 2001) used for this study were donated by Professor Weijia Kong of the Union Hospital Affiliated to Tongji Medical College, Huazhong University of Science and Technology. All experiments were performed on animals of both sexes following the guidelines approved by the Ethics Committee of Xinhua Hospital affiliated to Shanghai Jiaotong University School of Medicine.

#### Breeding and Tamoxifen Injection

To achieve time-conditional Cx26 deletion, we adopted a mating scheme previously used to generate mice with targeted ablation of Cx26 in the inner ear (Crispino et al., 2011; Fetoni et al., 2018). First, *Gjb2*^loxP/loxP^ mice were mated with ROSA26^CreER^ mice, yielding *Gjb2*^loxP//wt^; ROSA26^CreER^ mice (wt = wild type *Gjb2* allele). Next, *Gjb2*^loxP//wt^; ROSA26^CreER^ mice were mated with *Gjb2*^loxP/loxP^ mice to obtain *Gjb2*^loxP/loxP^; ROSA26^CreER^ mice. Finally, to promote deletion of the floxed alleles, P0 offspring were given a single intraperitoneal (i.p.) injection of tamoxifen (TMX, T5648-1G, Sigma–Aldrich, USA), at a dose of 100 mg/kg of body weight, as previously reported (Sun et al., 2009; Chang et al., 2015).

#### Mouse Genotyping

Mouse genotyping was performed as previously described (Chen et al., 2018). The primer pairs used to detect the loxP sequences were as follows:

forward 5’-CTTTCCAATGCTGGTGGAGTG-3’;

reverse 5’-ACAGAAATGTGTTGGTGATGG-3’.

*Gjb2*^loxP/loxP^ and wild-type mice generated a band of 322 bp and 288 bp, respectively.

Primer pairs used to detect the CreER sequences were as follows:

forward 5’-TATCCAGGTTACGGATATAGTTCATG-3’; and

reverse, 5’-AGCTAAACATGCTTCATCGTCGGTC-3’, which generated a band of 700 bp.

### Auditory Brainstem Response Test

Mice injected with tamoxifen at P0 were tested for auditory brainstem response (ABR) at P21 (Zhou et al., 2006). Six mice, three males, and three females were tested for each group. Animals were anesthetized with ketamine (120 mg/kg, i.p.) and chlorpromazine (20 mg/kg, i.p.) and placed in a sound-attenuating chamber on a heating pad to maintain body temperature. Tone burst stimuli were generated in the free field at frequencies of 4, 8, 16, and 32 kHz and amplitudes ranging from 0 to 100 dB sound pressure level (SPL) using a system equipped with the RZ6 hardware for data acquisition and sound production, Medusa4Z amplifier and MF1 multi-field magnetic speakers (TDT, Tucker-Davis Technologies, Alachua, FL, USA). Responses were amplified and averaged 512 times using the TDT BioSigRZ software.

### Immunohistochemistry

Mice injected with tamoxifen at P0 were used to obtain cochlear tissue at P21 after rapid decapitation. The cochlea was fixed with 4% paraformaldehyde overnight, decalcified with 10% EDTA, embedded in paraffin and sectioned, stained for immunohistochemistry with primary antibodies selective for Cx26 (PA518618, Invitrogen, USA) and Cx30 (700258, Invitrogen, USA), followed by incubation with HRP labeled secondary antibody (donkey anti-goat IgG, goat anti-rabbit IgG, Servicebio). Finally, samples were incubated with a DAB reaction kit (G1212-200T, Servicebio, China), which is the chromogenic substrate of HRP, and images were collected using a Nikon E100 with Nikon DS-U3 imaging system.

### Analyses of Cochlear Duct and Kölliker’s Organ Tissues

Mice were injected with tamoxifen at P0 and sacrificed by decapitation after 24 h to obtain cochlear duct and Kölliker’s organ tissues which were processed as described hereafter.

The cochlear duct was dissected in cold phosphate-buffered saline (PBS), fixed with 4% paraformaldehyde for 30 min, embedded in paraffin, and sectioned. A TUNEL detection kit (11684817910, Roche, Switzerland) was used to detect apoptosis in paraffin-embedded cochlear tissue sections following the manufacturer’s protocols.

For immunofluorescence staining, paraffin sections were incubated with primary antibodies selective for c-cas3, LC3-II, P62 (GB11009-1, Servicebio; ab192890, Abcam; GB11239-1, Servicebio) and a secondary antibody (Cy3-sheep-anti-rabbit, GB21303, Servicebio) respectively. Processed samples were observed and imaged with a fluorescence microscope (Nikon ECLIPSE CI with Nikon DS-U3 imaging system) under uniform illumination and detection conditions. The excitation wavelength was 550 nm and the emission wavelength was 570 nm.

For Western blot analyses, tissues were dissected in cold PBS and frozen in liquid nitrogen immediately after decapitation. The total protein content of the cochlea was extracted in RIPA lysis buffer (Servicebio, Wuhan, China) and quantitated following the kit instructions (BCA Protein Assay Kit, Beyotime, Haimen, China). The same amount of protein (20 μg per lane) was electrophoresed in a 15% sodium dodecyl sulfate-polyacrylamide gel and transferred to polyvinylidene difluoride (PVDF) membranes. After blocking with TBST containing 5% skimmed milk for 1 h, the sample was incubated at 4°C overnight with the primary antibodies selective for GAPDH (60004-1-lg, PTG), caspase 3 (66470-2-lg, PTG), Bcl-2 (GB13458, Servicebio), LC3 (GB11124, Servicebio), p62 (18420-1-AP, PTG), Beclin1 (GB112053, Servicebio). Next, samples were incubated at room temperature with horseradish peroxidase (HRP)-conjugated secondary antibody (GB23301, GB23303, Servicebio) for 1 h. The ECL reaction buffer (G2014, Servicebio) was added to detect the proteins in a Chemidoc XRS+ imaging system (BioRad, CA, USA).

For Luciferin–luciferase ATP bioluminescence assay, the cochlea was removed after rapid decapitation, the bony wall and the membranous labyrinth were separated from the apex to the base of the cochlea and the cochlear duct was dissected in cold phosphate-buffered saline (PBS). Next, the sensory epithelium was separated from the spiral ligament, Kölliker’s organ was micro-dissected and placed in a lysis buffer (S0027, Beyotime, China) and lysed in a Polytron PT1200 homogenizer (Kinematica, Luzern, Switzerland). Finally, the total ATP concentration was measured with a luciferin-luciferase bioluminescence ATP assay kit (S0027, Beyotime, China) using the Chemidoc XRS+ imaging system. All measurements reported in this article fell within the linearity range of the ATP standard curve generated according to the manufacturer’s instructions. All experiments were performed at room temperature (22–25°C).

### Preparation of Kölliker’s Organ Cultures From P0 Pups

For these experiments, we used pups from the same litter which was reserved for their tails for genotype identification during the experiment.

Kölliker’s organ was micro-dissected in cold 1× Hank’s balanced salt solution (HBSS, Thermo Fisher, 14025076, USA) as described above, transiently transferred to an Eppendorf tube containing DMEM/F12 mixed with 2% ampicillin (ST008, Beyotime, China) and then cultured as previously reported (Chen et al., 2018). Briefly, Kölliker’s organ was divided into three fragments from apex to base and the fragments were placed in a 24-well plate containing a tissue culture-treated, round glass slide (14 mm diameter, WHB-24-CS, WHB) immersed in DMEM/F12 containing 1% ampicillin, 10% fetal bovine serum (10099-141, Gibco, Australia). Alternatively, for Ca^2+^ imaging experiments with fluo-4 (see below), Kölliker’s organ fragments were placed on a tissue culture-treated glass-bottomed culture dish (801001, NEST, China). In either case, the culture medium was supplemented with 10 μM (Z)-4-hydroxytamoxifen (H7904, Sigma, Germany) to promote Cre recombinase-mediated *in vitro* excision of the floxed Cx26 alleles. Finally, samples were placed in an incubator (Thermo Scientific Forma Direct Heat CO_2_ Incubators) and cultured at 37°C, 5% CO_2_ for 12 h (Chen et al., 2018).

### Visualization of ATP-Loaded Vesicles in Kölliker’s Organ Cultures

Kölliker’s organ cultures, prepared as described above from P0 pups, were treated with quinacrine dihydrochloride (5 × 10^−6^ mol/L, orb320518, Biorbyt, UK) in 1× PBS solution for 30 min in the dark, at room temperature, washed three times with PBS, fixed with 4% paraformaldehyde for 1 h, washed three more times with PBS, incubated with cell permeabilizing solution (0.1% Triton X-100 in PBS) for 20 min and blocking solution (10% donkey serum in PBS) for 1 h, washed three times with PBS for 5 min each, and incubated overnight with an anti-LAMP1 primary antibody—lysosome marker (ab208943, Abcam). The next day, Kölliker’s organ cultures were removed from the primary antibody incubation solution, washed three times with PBS for 5 min each, and incubated with a secondary antibody (donkey anti-rabbit IgG, AlexaFluor 594, R37119, Invitrogen) at room temperature for 2 h, washed three times with PBS for 5 min each, and incubated with 4’, 6-diamidino-2-phenylindole (DAPI, D9542, Sigma) nuclear staining solution for 8 min. Stained cultures were mounted in an antifade mounting medium (H-1200-10, Vectorlabs, USA) and imaged with a confocal microscope (TCS-SP8, Leica, Germany). Fluorescence images of quinacrine (green), DAPI (blue), and LAMP1 (red) were obtained with a ×63 oil immersion objective (Leica) at excitation wavelengths of 488 nm, 405 nm, and 594 nm, respectively. The corresponding emission wavelengths were centered around 520 nm, 422 nm, and 617 nm, respectively.

### Ca^2+^ Imaging With Fluo-4 in Kölliker’s Organ Cultures

Kölliker’s organ cultures, prepared as described above from P0 pups, were incubated for 20 min at 37°C in 4 μM fluo-4 AM loading solution (F14201, Thermo Fisher) containing 20% Pluronic F-127, mixed with five times volume of HBSS (14025076, Thermo Fisher) containing 1% fetal bovine serum, incubated for further 40 min at 37°C, washed with HEPES buffer (10 mM HEPES, 1 mM Na_2_HPO_4_, 137 mM NaCl, 5 mM KCl, 1 mM CaCl_2_, 0.5 mM MgCl_2_, 5 mM glucose, 0.1% BSA, pH 7.4) three times, resuspended in HEPES buffer, and incubated for another 10 min at 37°C to allow baseline Ca^2+^ levels to stabilize. Using a spinning-disk confocal microscope (Nikon CSU-W1, Japan) with excitation and emission wavelengths set at 494 nm and 516 nm, respectively, we first imaged the baseline fluorescence intensity of fluo-4 for 2 min. Thereafter, while continuing image collection, we replaced the incubation solution with a HEPES buffer supplemented with 30 μM ATP (A6559, Sigma–Aldrich, USA) to stimulate purinergic receptors of Kölliker’s organ cells.

For off-line data analysis, single-pixel intensity values were background-subtracted and spatially averaged over regions of interest (ROIs) corresponding to individual cell bodies. Time-dependent fluctuations of intracellular Ca^2+^ levels were represented through the ratio *F*/*F*_0_, where *F* is the ROI signal at time *t* and *F*_0_ is the time-averaged pre-stimulus ROI intensity value (Mammano and Bortolozzi, 2010). *F*_max_ denotes the peak Ca^2+^-dependent fluorescence intensity fluctuation within a given ROI during each recording period (7 min in total). Data were computed as mean ± standard deviation of *n* = 30–50 cells from three separate experiments.

### Statistical Analysis

Statistical analysis of experimental data was performed with GraphPad Prism v8.0 (GraphPad Software, Inc., CA, USA) and Student’s t-test. P = p-values less than 0.05 were considered statistically significant.

## Results

### Severe Hearing Loss and Decreased Expression of Cx26 in the Cochlea of *Gjb2*^loxP/Loxp^; ROSA26^CreER^ Mice Injected With Tamoxifen at P0

**Figure 1:**
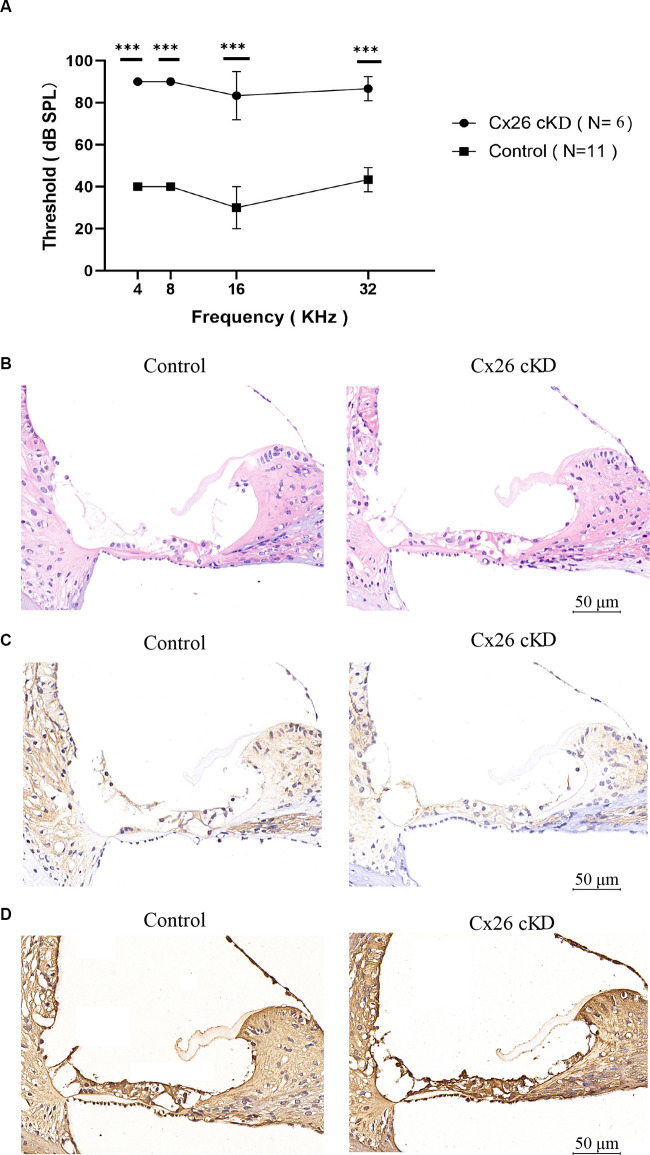
Effect of tamoxifen injection at P0 on the auditory threshold and connexin expression at P21. **(A)** Auditory thresholds measured by pure tone ABRs vs. tone frequency; ***p < 0.0001. **(B–D)** Transverse sections of the cochlear duct stained with hematoxylin-eosin **(B)** or with antibodies selective for Cx26 **(C)** or Cx30 **(D)**. Scale bars = 50 μm.

At P21, ABR results showed severe hearing loss in *Gjb2*^loxP/loxP^; ROSA26^CreER^ mice that had been injected with tamoxifen at P0 (shortened as Cx26-cKD mice). Average hearing thresholds in these mice (*n* = 6) exceeded 80 dB at 4, 8, 16 and 32 kHz and were significantly more elevated (*P* < 0.01) than thresholds of other genotypes injected with TMX at P0 and used as controls (*Gjb2*^loxP/wt^; ROSA26^CreER^, *n* = 5; *Gjb2*^loxP/loxp^, *n* = 3; *Gjb2*^loxP/wt^, *n* = 3; Figure 1A). In addition, immunohistochemical staining revealed a collapsed organ of Corti with an almost invisible cochlear tunnel (Figure 1B) and a decreased expression of Cx26 in supporting cells of the organ of Corti, in epithelial cells of the inner sulcus and outer sulcus, in the spiral limbus, among fibrocytes of the lateral wall and in the basal cell region of the *stria vascularis* of Cx26-cKD mice (Figure 1C), whereas expression of Cx30 was increased (Figure 1D).

### Abnormal Apoptosis and Autophagy in Kölliker’s Organ of Cx26-cKD Mice

**Figure 2:**
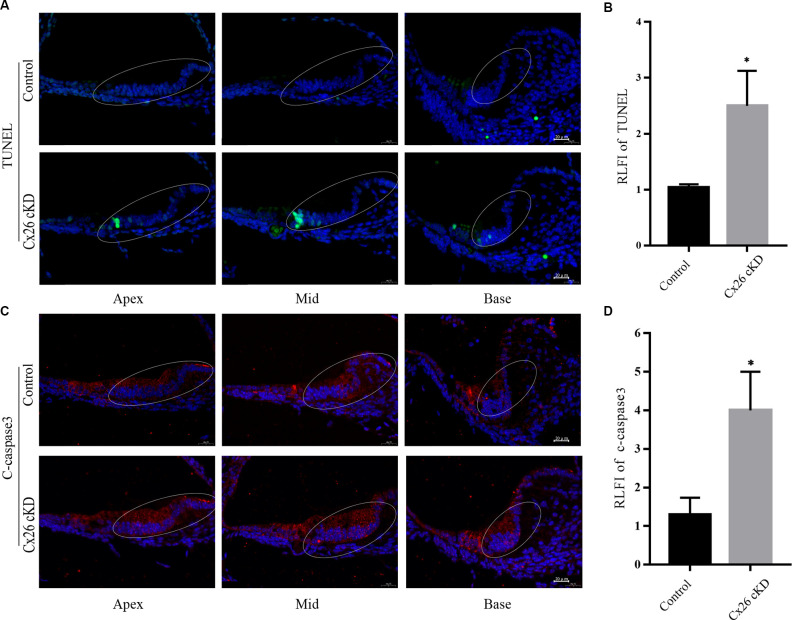
Apoptosis of Kölliker’s organ was detected by immunofluorescence staining at P1. **(A)** Representative confocal fluorescence images showing TUNEL positive cells (green) in Kölliker’s organ of the Cx26-cKD group, but not in the control group. Blue signals correspond to cell nuclei stained with DAPI. Scale bars = 50 μm. **(B)** Average relative fluorescence intensity (RLFI) of TUNEL signals; * represent *p* < 0.05, *n* = 3. **(C)** Immunofluorescence staining of cleaved (c)-caspase 3 in the apical, middle, and basal turns of Kölliker’s organ cells of Cx26-cKD group compared with the control group. Scale bars = 50 μm. **(D)** Average relative fluorescence intensity (RLFI) of the c-caspase 3 signal; * represents *P* < 0.05, *n* = 3.

As mentioned in the introduction, prior work with mouse models indicate that Cx26 expression has a profound impact on the development of the cochlear sensory epithelium through a complex interplay between Ca^2+^ signaling, autophagy, and apoptosis; reviewed in Mammano and Bortolozzi (2018) and Mammano (2019). Therefore, we harvested the cochleae of Cx26-cKD mice at P1 and used a TUNEL assay to visualize apoptotic cells (Gorczyca et al., 1993) in 8 μm-thick transverse sections of the cochlear duct (Figures 2A,B). Positive cells (green) were observed exclusively in Kölliker’s organ of the Cx26-cKD group, with 8 ± 1 positive cells (green) adjacent to the pro-sensory domain region in all cochlear turns, whereas no TUNEL positive cells were detected in the control group (*n* = 3). At this developmental stage, there was no sign of apoptosis in IHCs and OHCs of either Cx26-cKD or control groups.

Activated caspase-3 and -7 convert other procaspases to activated caspases, leading to the amplification of the apoptosis cascade (Slee et al., 1999; Logue and Martin, 2008). Thus, we quantified caspase 3 levels by immunofluorescence at P1 and detected significantly enhanced immunoreactivity in Kölliker’s organ of all cochlear turns in the Cx26-cKD group compared to controls (Figures 2C,D, *P* < 0.05, *n* = 3).

**Figure 3:**
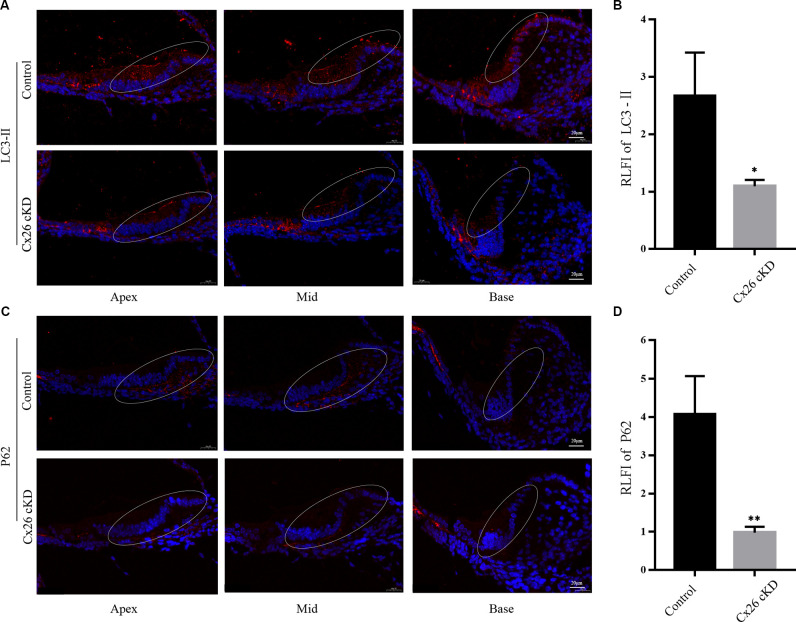
Autophagy of Kölliker’s organ was detected by immunofluorescence staining at P1. **(A)** In the apical, middle, and basal turns, immunoreactivity of LC3-II was intense in Kölliker’s organ of the control group and markedly less intense in the Cx26-cKD group. Scale bars = 50 μm. **(B)** Average relative fluorescence intensity (RLFI) of LC3-II signal; * represents *P* < 0.05, *n* = 3. **(C)** Immunofluorescence staining of p62 was attenuated in Kölliker’s organ cells of apical, middle, and basal turns in the Cx26-cKD group compared with the control group. Scale bars = 50 μm. **(D)** Average relative fluorescence intensity (RLFI) of p62; ** represents *P* < 0.001, *n* = 3.

LC3-II and p62 are widely used molecular markers of autophagy (Kabeya et al., 2000; Emanuele et al., 2020). At P1, we found diffused immunoreactivity against LC3-II in the sensory epithelium of the control group, with a peak in the pro-sensory region. In the Cx26-cKD group, immunoreactivity against LC3-II was significantly decreased in Kölliker’s organ (Figures 3A, B, *P* < 0.05, *n* = 3). Likewise, we found decreased immunoreactivity against p62 in Kölliker’s organ cells of the Cx26-cKD group compared to the control group (Figures 3C,D, *P* < 0.001, *n* = 3). Together, the results of Figures 2, 3 suggest that decreased expression of Cx26 leads to increased apoptosis and decreased autophagy in Kölliker’s organ at P1.

**Figure 4:**
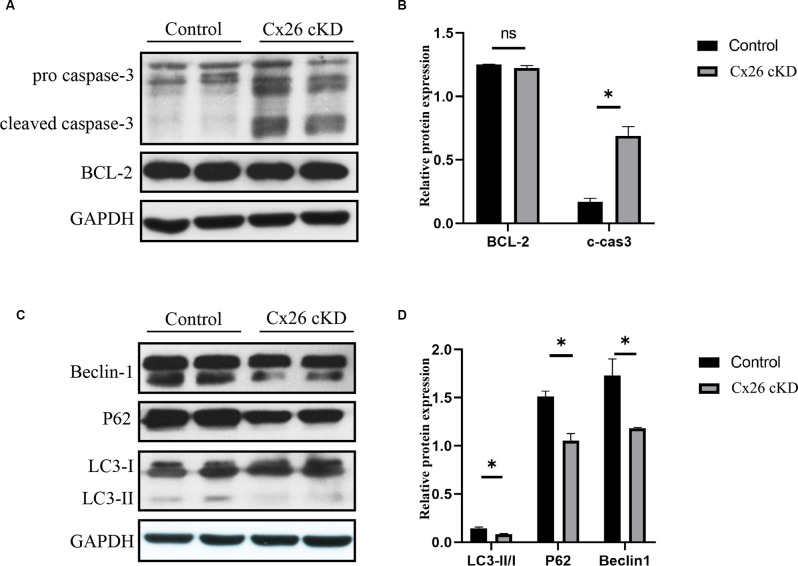
Western blot analysis of apoptosis- and autophagy-related proteins in Kölliker’s organ at P1. **(A)** Representative western blots for apoptosis-related proteins Bcl-2 and c-caspase 3. **(B)** Corresponding relative optical density; * represents *P* < 0.05, *n* = 5. **(C)** Representative western blots for autophagy-related proteins LC3-II, P62 and Beclin1. **(D)** Corresponding relative optical density; * represents* P* < 0.05, *n* = 5.

To corroborate this conclusion, we investigated expression levels of apoptosis and autophagy markers by Western blotting (Figure 4). At P1, we confirmed that cleaved caspase-3 was upregulated in the Cx26-cKD group, with a significant difference compared with the control group (*P* < 0.05, *n* = 5; Figures 4A,B). However, these alterations did not involve the anti-apoptotic factor Bcl-2 (Vaux et al., 1992), which is expressed from the 15th day of embryonic development (E15) to P5 in the normal mouse cochlea (Ishii et al., 1996; Kamiya et al., 2001). Our Western blot analyses showed no statistically significant differences (*P* > 0.05, *n* = 5) in the expression level of Bcl-2 between the Cx26-cKD and control mice at P1 (Figures 4A,B).

In the normal mouse cochlea, beclin1 and other autophagy-related proteins start to be expressed in the late embryonic stage and continue to be upregulated after birth, until the inner ear achieves functional maturity of the adult stage (de Iriarte Rodriguez et al., 2015). In the Cx26-cKD group, beclin1, LC3-II/I, and p62 were significantly downregulated compared with the control group (*P* < 0.05, *n* = 5; Figures 4C,D). Together, these results suggest that in Kölliker’s organ of Cx26-cKD mice at P1, downregulation of autophagy is accompanied by the upregulation of apoptosis independent of Bcl-2 expression.

### Decreased Total ATP Content in Kölliker’s Organ of Cx26-cKD Mice

Prior work showed that decreased levels of Cx26 expression in the mouse postnatal cochlea reduce gap junction coupling, limiting the transfer of nutrients, and glucose in particular, from distant blood vessels to the avascular sensory epithelium (Fetoni et al., 2018). Upon glucose deprivation, autophagy is induced to supplement the metabolic pool and provide ATP through various mechanisms (Galluzzi et al., 2014). However, this process is hampered if autophagy is downregulated, therefore we predicted an overall reduced intracellular ATP concentration downstream of Cx26 knockdown.

**Figure 5:**
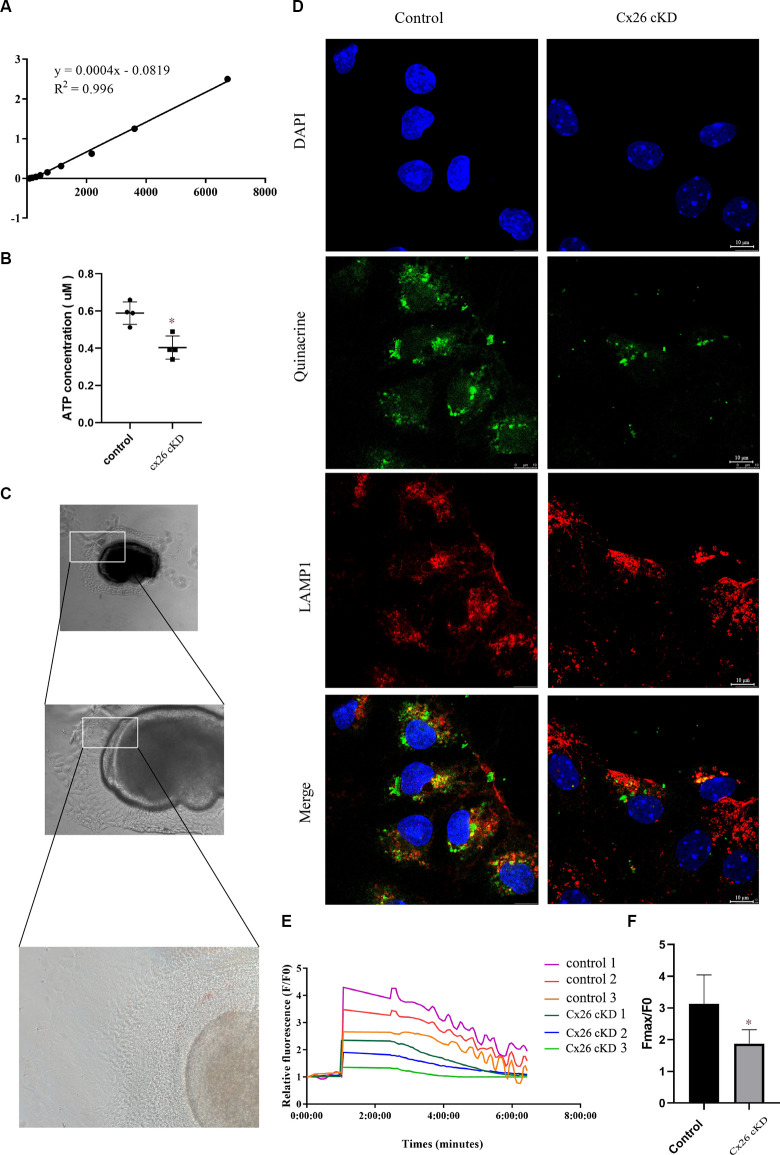
ATP concentration and ATP-evoked Ca^2+^ signaling. **(A)** Representative standard curve used to calibrate bioluminescence signals: *Y* = 0.0004X − 0.0819, *R*^2^ = 0.996. **(B)** ATP concentration estimated from luciferin-luciferase bioluminescence assay. *P* < 0.05, *n* = 4. **(C)** Representative transmitted light microscopy image of a Kölliker’s organ culture viewed at 10×, 20×, and 40× magnification (from top to bottom). **(D)** Representative confocal fluorescence images of Kölliker’s organ cultures labeled with quinacrine, anti-LAMP1 antibodies, and DAPI; scale bars = 10 μm. **(E)** Representative ATP-evoked Ca^2+^ responses of individual cells in Kölliker’s organ cultures. **(F)** Quantification of *F*_max_/*F*_0_ signals; * represents *P* < 0.05, *n* = 3.

To test this hypothesis, Cx26-cKD mice and their controls were sacrificed at P1. The micro-dissected Kölliker’s organ was lysed and the total ATP concentration in the lysate was measured with a luciferin-luciferase bioluminescence ATP assay kit. To minimize the experimental changes, a standard curve was constructed for each experiment to estimate the corresponding ATP concentration (Figure 5A). ATP levels in the Cx26-cKD group were significantly reduced compared with the control group (Figure 5B; *P* < 0.05, *n* = 4), confirming our hypothesis.

### Decreased Number of ATP-Loaded Vesicles in Kölliker’s Organ of Cx26-cKD Mice

The vesicular nucleotide transporter, also known as solute carrier family 17 member 9 (SLC17A9), mediates lysosomal ATP accumulation and plays an important role in lysosomal physiology and cell viability (Cao et al., 2014). Based on the results described above, we predicted that the overall reduced ATP availability should correspond to a lower amount of ATP in the lysosomes of Kölliker’s organ cells, where prior work showed ATP is accumulated (Chen et al., 2019).

To test this hypothesis, we prepared Kölliker’s organ cultures from untreated P0 pups of *Gjb2*^loxP/loxP^; ROSA26^CreER^ mice, and control mice. To promote Cre recombinase-mediated *in vitro* excision of Cx26 floxed alleles, cultures were exposed to 10 μM (Z)-4-hydroxytamoxifen (HTMX) and thereafter inspected by transmitted light microscopy at 10, 20, and 40× magnification (Figure 5C). No visible differences were noted between the HTMX and control groups. Therefore, we proceeded to stain the cultures with DAPI (to label nuclei) and quinacrine, which functions as an ATP-binding agent and acridine derivative with a very high affinity to ATP and has been used to label ATP-containing vesicles (White et al., 1995; Chen et al., 2019). Samples were also immuno-stained with an anti-LAMP1 primary antibody (a lysosome marker) and a suitable secondary antibody (see “Materials and Methods” Section). The quinacrine signal (green) in the HTMX group was lower than in the control group, whereas LAMP1 immunoreactivity (red) was not significantly different between the two groups (Figure 5D). These qualitative results accord with the quantitative results of Figure 5B and suggest that, as a consequence of Cx26 knockdown, less ATP was accumulated in lysosomal vesicles of the Cx26-cKD group compared with the control group.

### Decreased ATP-Evoked Intracellular Ca^2+^ Responses in Cx26-cKD Cochlear Cultures

To determine whether the alterations described above affect also Ca^2+^ signaling, HTMX-treated Kölliker’s organ cultures were loaded with the selective Ca^2+^ indicator fluo-4 and challenged by the application of saturating amounts of exogenous ATP (30 μM, see “Materials and Methods” Section), expected to cause massive Ca^2+^ release from the ER. Ca^2+^ imaging revealed peak responses (*F*_max_/*F*_0_) in the Gjb2^loxP/loxP^; ROSA26^CreER^ group that were significantly downregulated compared with the control group (1.86 ± 0.37 vs. 3.13 ± 0.77, *P* < 0.05, *n* = 3; Figure 5F). In addition, we noted that Ca^2+^ oscillations appeared during the declining phase of the responses in the control group but were absent in the Gjb2^loxP/loxP^; ROSA26^CreER^ group (see “Discussion” Section, for a possible explanation). Together, the results in Figure 5, show that the knockdown of Cx26 affects a major ATP-dependent Ca^2+^ signaling pathway in Kölliker’s organ, which is crucial for organ development and hearing acquisition as summarized in the introduction.

## Discussion

The organ of Corti is the core part of the auditory system, composed of hair cells and supporting cells. The hair cells function in transducing the sound mechanical stimulation into the primary acoustic signals (Liu et al., 2019), while the spiral ganglions transmit primary acoustic information from hair cells in the organ of Corti to the higher auditory centers of the central nervous system (Wei et al., 2021). Hair cells are easily injured by excessive noise exposure (Guo et al., 2021; He et al., 2021), ototoxic drugs (He et al., 2017), aging (He et al., 2020), genetic factors (Fu et al., 2021), and infections (He et al., 2020).

Mouse models continue to provide critical insight into the functioning of the auditory system and deafness-associated genes (Bowl et al., 2017). Among these, it has long been known that Cx26 (*Gjb2*) biallelic deletion in mice is embryonically lethal due to impaired transplacental uptake of glucose (Gabriel et al., 1998). The *Gjb2*^loxP/loxP^ mice used in this study (Cohen-Salmon et al., 2002), can overcome embryonic lethality if crossed with a suitable Cre-expressing strain to achieve tissue- and/or time-conditional deletion of the floxed alleles (Orban et al., 1992; Vooijs et al., 2001). Crossing *Gjb2*^loxP/loxP^ mice with the Otog-Cre strain (Cohen-Salmon et al., 2002), or the Sox10-Cre strain (Anselmi et al., 2008) resulted in mice with severe hearing loss and developmental defects in the cochlear sensory epithelium (Cohen-Salmon et al., 2002; Crispino et al., 2011). Both *Gjb2*^loxP/loxP^; Otog-Cre and *Gjb2*^loxP/loxP^; Sox10-Cre mice are considered models of human DFNB1 non-syndromic hearing impairment, which is frequently associated with truncating mutations that yield non-functional Cx26 proteins (Chan and Chang, 2014; Del Castillo and Del Castillo, 2017).

Results obtained from time-dependent knockdown of Cx26 in tamoxifen-induced *Gjb2*^loxP/loxP^; ROSA26^CreER^ mice (Chang et al., 2015; Chen et al., 2018) lend further support to the notion that Cx26 intercellular gap junction channels and hemichannels with normal permeability to nutrients and other metabolites and signaling molecules are essential for normal development of the cochlea and normal hearing acquisition (Mammano, 2019).

Here, using tamoxifen-induced knockdown of Cx26 in *Gjb2*^loxP/loxP^; ROSA26^CreER^ mice, we found decreased levels of autophagy-related proteins beclin1, LC3-II, and p62 in the cochlea, indicating that autophagy was downregulated. These alterations were accompanied by increased apoptosis in the Kölliker’s organ cells, which became apoptotic as early as P1 based on TUNEL assays and upregulation of c-caspase 3 expressions. The p62 marker is particularly interesting because increasing evidence points to the N-terminal arginylated BiP (R-BiP)/Beclin-1/p62 complex as having an important role in the crosstalk between apoptosis and autophagy, which greatly affects cell death (Song et al., 2018). Recent work examined this interplay in the normal developing cochlea and concluded that autophagy precedes apoptosis in the natural postnatal degeneration of Kölliker’s organ cells and their regulated replacement by cuboidal cells of the inner sulcus (Hou et al., 2019).

The accelerated apoptosis described in this article is easily explained in the light of a recent study that linked decreased Cx26 expression to apoptosis *via* impaired nutrient delivery to the sensory epithelium through gap junction channels, the reduced release of the key antioxidant glutathione through connexin hemichannels, and deregulated expression of several genes under the transcriptional control of Nrf2, a redox-sensitive transcription factor that plays a pivotal role in oxidative stress regulation (Fetoni et al., 2018; Ding et al., 2020). Thus, we conclude that impairment of Cx26 function subverts the critically timed phasing of autophagy and apoptosis in the mouse postnatal cochlea, hijacks the hearing acquisition program, and dooms animals to deafness through increased oxidative stress. This conclusion is also supported by prior studies showing that Kölliker’s organ cells did not completely degenerate until 2 weeks after birth in caspase 3 knockout mice, resulting in hyperplasia of supporting cells, degeneration of hair cells, and severe hearing loss (Takahashi et al., 2001), strengthening the notion that a correctly executed postnatal apoptotic program is key to hearing acquisition in mice (Chen et al., 2020).

In non-sensory cells of the cochlear sensory epithelium, ATP binding to G protein-coupled P2Y receptors activates the production of IP_3_ *via* phospholipase C (PLC), promoting Ca^2+^ release from the endoplasmic reticulum (ER) through IP_3_ receptors (IP_3_R) and consequent increase of the cytoplasmic free Ca^2+^ concentration (Mammano, 2013). Our Ca^2+^ imaging experiments show that the ATP/P2Y/PLC/IP_3_ signal transduction cascade, which fuels Ca^2+^ signaling in Kölliker’s organ, is downregulated by the knockdown of Cx26. To interpret these results, it is imperative to consider that: (i) all else held equal, the amount of Ca^2+^ released from the ER depends on the Ca^2+^ concentration in the ER; (ii) increased oxidative stress is associated with Cx26 downregulation in the developing cochlea (Fetoni et al., 2018; He et al., 2019). Thus, in our experimental conditions, SERCA pumps activity was lowered not only by the reduced availability of cytosolic ATP (this article) but also by the effect of oxidative stress (Kaplan et al., 2003). In addition, alterations in the redox state of critical thiols in the IP_3_R lead to sensitization of IP_3_R-mediated Ca^2+^ release associated with oxidative stress (Joseph et al., 2018), which may increase the steady-state Ca^2+^ leakage from the ER. The predicted net effect is a reduced Ca^2+^ content in the ER, hence a reduced driving force for Ca^2+^ transfer from the ER to cytosol driven by the signal transduction cascade mentioned above. This explains the reduced *F*_max_/*F*_0_ signals evoked by supramaximal exogenous ATP stimuli in Kölliker’s organ cultures exposed to HTMX.

As for the issue of Ca^2+^ oscillations, data-driven computational modeling shows that they are governed by Hopf-type bifurcation and arise only within a limited range of extracellular ATP concentration through the interplay of IP_3_R-mediated Ca^2+^ release from the ER and SERCA pump-mediated Ca^2+^ re-uptake into the ER (Ceriani et al., 2016). In control Kölliker’s organ cultures, oscillations arose during the recovery phase from supramaximal stimulation, while the extracellular ATP concentration lowered due to diffusion and ATP hydrolysis mediated by ectonucleotidases expressed at the surface of the epithelium (Ceriani et al., 2016). As both SERCA pump activity and IP_3_R are affected by oxidative stress, it comes as no surprise that Ca^2+^ oscillations were absent in Kölliker’s organ cultures exposed to HTMX. This conclusion is supported also by experiments and mathematical modeling of the effects of oxidative stress on Ca^2+^ oscillation in other cellular systems (Antonucci et al., 2015).

In conclusion, our results provide further evidence for abnormal cochlear development in mice with reduced expression of Cx26, expound possible mechanisms of hearing acquisition failure, and produce novel insight, from a new perspective, for *GJB2*-related hereditary deafness.

## Data Availability Statement

The raw data supporting the conclusions of this article will be made available by the authors, without undue reservation.

## Ethics Statement

The animal study was reviewed and approved by Ethics Committee of Xinhua Hospital affliated to Shanghai Jiaotong University School of Medicine.

## Author Contributions

LS and DG wrote the article. JuC, SH, YL, and YH analyzed the data. FM, JiC, and JY designed the study. All authors contributed to the article and approved the submitted version.

## Conflict of Interest

The authors declare that the research was conducted in the absence of any commercial or financial relationships that could be construed as a potential conflict of interest.

## Publisher’s Note

All claims expressed in this article are solely those of the authors and do not necessarily represent those of their affiliated organizations, or those of the publisher, the editors and the reviewers. Any product that may be evaluated in this article, or claim that may be made by its manufacturer, is not guaranteed or endorsed by the publisher.

## Abbreviations

KO: Kölliker’s organ
IHCs: inner hair cells
Cx26: gap junction protein beta-2
Cx30: gap junction protein beta-6.
